# Assessment of feigned cognitive impairment in severe traumatic brain injury patients with the Forced‐choice Graphics Memory Test

**DOI:** 10.1002/brb3.593

**Published:** 2016-10-13

**Authors:** Zilong Liu, Juan Dong, Xiaohong Zhao, Xiaorui Chen, Sara M. Lippa, Jerome S. Caroselli, Xiang Fang

**Affiliations:** ^1^ Department of Forensic MedicineTongji Medical CollegeHuazhong University of Science and TechnologyWuhanHubeiChina; ^2^ Defense and Veterans Brain Injury CenterWalter Reed National Military Medical CenterBethesdaMDUSA; ^3^ Department of Psychology/NeuropsychologyTIRR Memorial Hermann HospitalHoustonTXUSA; ^4^ Department of NeurologyUniversity of Texas Medical BranchGalvestonTXUSA

**Keywords:** malingering, performance validity, response bias

## Abstract

**Introduction:**

The Forced‐choice Graphics Memory Test (FGMT) is a newly developed measure to assess feigned cognitive impairment. This study investigated the ability and reliability of FGMT for identification of malingering in patients with traumatic brain injury (TBI).

**Methods:**

The FGMT was administered to 40 healthy volunteers instructed to respond validly (Healthy Control, H‐C), 40 healthy volunteers instructed to feign cognitive impairment (Healthy Malingering, H‐M), 40 severe TBI patients who responded validly (TBI control, TBI‐C), and 30 severe TBI patients who evidenced invalid performance (TBI malingering, TBI‐M).

**Results:**

Both malingering groups (H‐M and TBI‐M) performed much more poorly than the nonmalingering groups (H‐C and TBI‐C). The FGMT overall total score, score on easy items, and score on hard items differed significantly across the four groups. The total score showed the highest classification accuracy in differentiating malingering from nonmalingering. A cutoff of less than 18 (total items) successfully identified 95% of TBI‐C and 93.3% of TBI‐M participants. The FGMT also demonstrated high test–retest reliability and internal consistency. FGMT scores were not affected by TBI patients' education, gender, age, or intelligence.

**Conclusion:**

Our results suggest that the FGMT can be used as a fast and reliable tool for identification of feigned cognitive impairment in patients with TBI.

## 1 Introduction

In the *diagnostic and statistical manual of mental disorders, fifth edition*, malingering means that people are faking or really embellishing physical or psychological symptoms. People who are malingering do this “consciously” because there is an external incentive to do so (e.g., avoiding military duty or work, obtaining financial compensation, evading criminal prosecution, or obtaining drugs; American Psychiatric Association, 2013). Recently, malingering is one of the main issues presented in neuropsychological literatures (Sweet, King, Malina, Bergman, & Simmons, 2002; Vilar‐Lopez et al., 2007).

Traumatic brain injury (TBI) can result in cognitive impairment (Jennekens, de Casterle, & Dobbels, 2010; Miotto et al., 2010). Typically, neuropsychological evaluation is used to characterize the level and pattern of cognitive impairment for those who have sustained a TBI (Finnanger et al., 2013; Hellawell, Taylor, & Pentland, 1999; Satz et al., 1998). TBI is often the result of accidents that may involve litigation or secondary gain (Sweet, Goldman, & Guidotti Breting, 2013). These factors have been shown to influence the validity of one's performance on neuropsychological tests (Gouse, Thomas, & Solms, 2013). Numerous studies have shown that lower neuropsychological performance in patients with TBI may, in many cases, be accounted for by invalid effort rather than low ability (Flaro, Green, & Robertson, 2007; Green, Iverson, & Allen, 1999). Therefore, it is especially important to assess performance validity in TBI patients. It is estimated that 40%–60% of patients with TBI may malinger cognitive impairments during disability compensation evaluations (Larrabee, 2005; Mittenberg, Patton, Canyock, & Condit, 2002).

A variety of performance validity tests (PVTs) are available to assess poor effort or response bias (Frazier, Youngstrom, Naugle, Haggerty, & Busch, 2007; Hiscock & Hiscock, 1989; Vagnini, Berry, Clark, & Jiang, 2008; Van Dyke, Millis, Axelrod, & Hanks, 2013; Wisdom, Brown, Chen, & Collins, 2012). These PVTs include both formal tests developed specifically for the purpose of detecting poor effort as well as measures that are embedded into other neuropsychological tests. Tests of effort can also be separated into “forced‐choice” versus nonforced‐choice. Forced‐choice effort measures were originally developed with the idea that if a patient performed significantly below chance, they must know the correct answer and intentionally choose the wrong answer, indicating a purposeful attempt to appear more impaired than they actually are. Vickery and Berry conducted a meta‐analysis on PVTs (Vickery, Berry, Inman, Harris, & Orey, 2001). In their analysis, the Hiscock Digit Memory Test (DMT), which is based on a binomial forced‐choice paradigm, had the highest sensitivity to detect poor effort as well as the best overall classification rates. In another meta‐analysis published by Sollman and Berry (2011), Victoria Symptom Validity Test, which is a computerized version of forced‐choice DMT, was employed as “an anchor” to evaluate the utility of the previously reviewed tests by Vickery and Berry.

Forced‐choice tests were first described by Binder and Pankratz (1987). They presented a case of a 53‐year‐old woman who was suspected of invalid responding. They gave her the task of indicating whether a black pen or a yellow pencil had been presented in the prior trial. The patient was accurate for 37 of the 100 trials, which was significantly worse than chance, and consistent with the assertion of her responding invalidly. Based on Pankratz's test, Hiscock and Hiscock (1989) modified the procedure and developed the DMT. In this test, patients are asked to identify which of two five‐digit numbers is identical to a number shown seconds earlier. The test is divided into three segments, each with a different delay length: 5, 10, and 15 s. The patient is informed about the increase in delay time and it is suggested that this will make the task more difficult. Although DMT was developed to appear more difficult than other versions of forced‐choice tests designed to identify feigning of a memory deficit, it is so simple that very few malingerers will perform at below chance levels. Research has been conducted with forced‐choice tests to determine alternate criteria to distinguish malingerers from nonmalingerers such as performance‐level criteria (Loring, Larrabee, Lee, & Meador, 2007). Guilmette, Hart, Giuliano, and Leininger (1994), for example, determined that a score below 90% correct on the DMT is consistent with invalid performance. The Test of Memory Malingering (TOMM; Tombaugh, 1996), a forced‐choice visual recognition memory test, is another commonly used PVT for detection of malingering in forensic setting (Slick, Tan, Strauss, & Hultsch, 2004). The TOMM also yields great classification ability for detecting insufficient effort (Love, Glassmire, Zanolini, & Wolf, 2014; O'Bryant & Lucas, 2006).

There is abundant research evidence that cognitive effort tests are extremely useful, especially in forensic settings (Fox, 2011; Green, 2007; Green, Rohling, Iverson, & Gervais, 2003; Green, Rohling, Lees‐Haley, & Allen, 2001). However, there are still some unresolved issues that need to be addressed, such as the influence of the patient's intellectual level and psychiatric status on PVTs (Avila et al., 2009; Shandera et al., 2010). It has also been noted that patients with TBI may have difficulty with PVTs for a myriad of other reasons, including education level, attention impairments, or receptive language impairments (Schroeder, Twumasi‐Ankrah, Baade, & Marshall, 2012; Woods et al., 2011).

In instances where litigation is involved, lawyers may instruct their clients about PVTs (Lezak, Howieson, Bigler, & Tranel, 2012). Studies have investigated the effect of coaching on various types of malingering tests, such as Computerized Assessment of Response Bias‐97 (CARB‐97) and Word Memory Test (Dunn, Shear, Howe, & Ris, 2003), Rey's 15‐Item Test and Dot Counting Test (Erdal, 2004), Portland Digit Recognition Test (Gunstad & Suhr, 2001), Category Test (DiCarlo, Gfeller, & Oliveri, 2000), Medical Symptom Validity Test and the Amsterdam Short‐Term Memory Test (Merten, Green, Henry, Blaskewitz, & Brockhaus, 2005), and Short‐Term‐Memory Test from the Bremer Symptom‐Validierung (Russeler, Brett, Klaue, Sailer, & Munte, 2008). Coached malingering was difficult to detect using these various tests. Coached malingerers, especially those coached with the symptom plus test information, were more likely to be misclassified as nonmalingerers than uncoached malingerers. The Internet also provides an easy way for patients to gain familiarity with PVTs, making their noncredible performance harder to detect (Castiel, Alderman, Jenkins, Knight, & Burgess, 2012; Frederick & Speed, 2007). Therefore, it is important to develop newer PVTs.

To this end, we developed the Forced‐choice Graphics Memory Test (FGMT). The FGMT utilizes the two‐alternative forced‐choice paradigm and consists of figural stimuli, which are supposed to be more on an intuitive basis than digits and not susceptible to aforementioned factors such as intellectual level and educational experience (Chakrabarti & Banerjee, 2013; Mungkhetklang, Crewther, Bavin, Goharpey, & Parsons, 2016; Paivio, 1971). This study evaluates the usefulness of the FGMT for identifying valid and invalid performances.

## 2 Methods

### 2.1 Participants

The study included four groups of right‐handed participants. The first group consisted of 40 college students and staff members who were instructed to give their best effort (Healthy Control, H‐C). The second group consisted of 40 college students and staff members who were instructed to feign invalid responding (Healthy Malingering, H‐M). The participants of both healthy groups were recruited from Tongji Medical College of Huazhong University of Science and Technology via advertisement. Medical records of these healthy participants were collected, excluding those with psychiatric or neurologic disorders. The H‐M participants were instructed to imagine that they sustained a TBI as a result of a motor vehicle accident 6 months ago and were currently involved in litigation to obtain financial compensation for their injury. They were told that an associated neuropsychological evaluation was going to take place and worse performance on the tests would contribute to a greater amount of injury compensation. They were additionally told to feign the TBI symptoms of headache, dizziness, hypomnesia, or unresponsiveness and to get low scores on tasks by reduced engagement in the tasks or by providing incorrect response. All of the TBI participants were recruited from a forensic medicine clinic of Tongji Medical College. They were referred for forensic evaluations (i.e., litigation, compensation seeking, or disability) and met the following inclusion criteria: (1) The TBI was sustained 6–12 months prior to participation in the study with clinical treatment having been completed; (2) Positive brain imaging findings of brain injury; (3) The lowest recorded Glasgow Coma Scale (Teasdale & Jennett, 1976) score in the first 6 hr on first admission without the presence of sedatives and paralytics in the range of 3–8; and (4) A negative preinjury history of psychiatric or neurologic disorders. A participant was specifically placed in the TBI Control (TBI‐C) group if they met these additional criteria: (1) Two forensic psychiatry experts conducting separate evaluations agreed that the person with TBI was presenting validly; and (2) the participant passed a forced‐choice measure, viz., the Binomial Forced‐Choice Digit Memory Test (BFDMT). The TBI‐C group consisted of 40 participants and they were instructed to give their best effort. The fourth group, TBI Malingering (TBI‐M) group, consisted of 30 participants who met the Slick criteria for Malingered Neurocognitive Dysfunction (MND; Slick, Sherman, & Iverson, 1999). The four criteria for the determination of MND are: (A) At least one clearly identifiable and substantial external incentive for exaggeration or fabrication of symptoms is present at the time of examination; (B) Evidence of exaggeration or fabrication of cognitive dysfunction on neuropsychological tests; (C) Significant inconsistencies or discrepancies in the patient's self‐reported symptoms that suggest a deliberate attempt to exaggerate or fabricate cognitive deficits; and (D) Behaviors meeting the necessary B or C criteria not fully accounted for by psychiatric, neuropsychological, or developmental disorders that result in significantly diminished capacity to appreciate laws or mores against malingering, or inability to conform behavior to such standards. On the basis of these four criteria, patients can be classified as: not malingering, definite MND, probable MND, or possible MND. In this study, only those patients who met the criteria of definite MND were recruited. Criterion B for this study was considered satisfied as all the members of the group showed a negative response bias on the BFDMT, that is, they each performed significantly below chance; Criterion C for this study was considered satisfied for each participant in this group as two forensic experts conducting separate evaluations agreed that the person with TBI was feigning due to the inconsistencies or discrepancies in participant's self‐report histories, and symptoms or performance across neuropsychological testing. The study protocol was approved by the Ethics Committee of Huazhong University of Science and Technology.

Informed consent was obtained from the participants after they had been given an explanation of the study. The individuals were informed that they would undergo several neuropsychological tests, and the data may be used for scientific analysis while maintaining their confidentiality.

### 2.2 Binomial Forced‐choice Digit Memory Test

The BFDMT is a PVT. Each participant completed the BFDMT, a revised version of the DMT developed by Liu, Gao, and Li, (2001). This test has been shown to have an overall accuracy of 95%, false‐positive rate of 1%, and false‐negative rate of 4% when healthy simulators were differentiated from healthy controls (Liu et al., 2001). The BFDMT is a commonly used PVT in China (Liu et al., 2001) and has been validated in different populations such as mental retardation, TBI, schizophrenia, and the elderly with cognitive impairment (Chu et al., 2010; Gao, Liu, Ding, Li, & Sheng, 2002; Gao, Yang, Ding, Li, & Sheng, 2003; Zhang, Liu, Chu, Li, & Chen, 2009). It is largely based on the binomial theorem. The test consists of 24 items, each of which consists of one stimulus card containing a single five‐digit number and a corresponding recognition card containing two five‐digit numbers. Each stimulus card is presented on the computer for 5 s, and is immediately followed by a recognition card. There are 12 easy items and 12 hard items based on the degree of similarity between the two five‐digit numbers presented on the recognition card. The more similar numbers comprised the hard items and the more different numbers comprised the easy items.

### 2.3 Forced‐choice Graphics Memory Test

**Figure 1:**
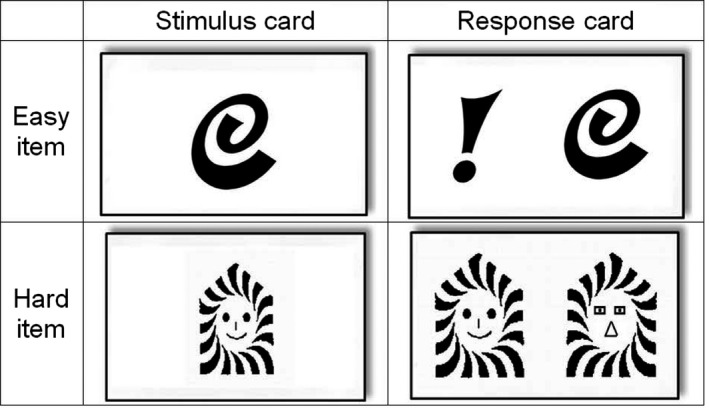
The sample cards of Forced‐choice Graphics Memory Test

The FGMT is a PVT that is modeled after the BFDMT. This task also consists of 24 items, each of which consists of one stimulus card and one corresponding recognition card. Each stimulus card contains one black and white design. Each design is round with a 6.5 cm diameter and 500 × 500 pixels. For each stimulus card, there is a corresponding recognition card containing two designs presented side‐by‐side. One of the designs matches the original design presented on the stimulus card (i.e., the target) and the other one is a distractor. The left‐or‐right side location of the target design was selected randomly. Each stimulus card was presented on a computer screen for 5 s, and was followed by the presentation of the corresponding recognition card after a 5 s retention period. The participants were asked to identify which design he or she had just viewed. According to the degree to which the two designs were similar on the recognition card, cards were classified as easy (i.e., less similar) or hard (i.e., more similar). Three of the authors ranked the similarity of 60 cards separately and classified into easy and hard cards. Then 12 easy cards and 12 hard cards that all of the three authors agreed were selected for the test. Sample items are shown in Figure 1. The order in which the cards were presented was random. Each item resulted in a score of 1 for a correct recognition of the target or 0 for an incorrect answer. Three scores were computed for each participant: total score, easy item score, hard item score. Test administration time was generally 5–10 min.

### 2.4 Wechsler Adult Intelligence Scale‐III Chinese version (WAIS‐RC)

Intellectual testing was carried out post‐PVT performance using the WAIS‐RC (Yao, Chen, Jiang, & Tam, 2007). The Verbal IQ, Performance IQ, and Full IQ were calculated for each participant.

### 2.5 Procedure

Demographic information and medical history were gathered for all participants. After completing the consent form, each participant was administered the BFDMT for classification. Then all participants were given instructions that varied by group. The H‐C group was asked to perform optimally during the tests. The H‐M group was given the information of TBI and post‐TBI cognitive impairment. This group was instructed to feign memory impairment during the tests for getting more compensation. After the instructions, other neuropsychological testing that, in part, included the FGMT and WAIS‐RC were administrated. For the TBI participants, an interview with two forensic psychiatrists was performed initially. In order to measure the reliability and consistency of FGMT, 20 TBI‐C and 20 TBI‐M participants completed the FGMT again a week later.

### 2.6 Statistical analysis

Data are expressed as mean ± *SD*. Correlation analysis between BFDMT total score and FGMT total score, easy item score, and hard item score were performed using Pearson's analysis. The sensitivity, specificity, predictive power, overall hit rate, and internal consistency for each of the FGMT indices were calculated. Comparisons of differences in FGMT indices were performed using Kruskal–Wallis one‐way ANOVAs followed by post hoc analysis for multiple groups.

## 3 Results

### 3.1 Characteristics of the participants

The brain injuries of TBI participants were caused by various events including traffic accidents (74%), falls (17%), assault (6%), and other reasons (3%). The majority of the patients presented with lesions in the frontal and temporal region (frontal lobes, 40%; temporal lobes, 21%; fronto‐temporal lobes, 17%). The remaining 22% presented with lesions in occipital and parietal region. Mann–Whitney test did not reveal significant differences in the duration of loss of consciousness (LOC) or post‐traumatic amnesia (PTA) between TBI‐C and TBI‐M group. The median duration of LOC in patients of TBI‐C and TBI‐M group was 8 days and 9 days, respectively (*p *=* *.91), and all the patients have a PTA of 24 hr or more (median 18 days and 15 days in TBI‐C and TBI‐M group, respectively, *p *=* *.39). None of the TBI participants in this study had received systematic cognitive rehabilitation following injury.

The demographic information for each group is presented in Table 1. Chi‐square analysis did not reveal significant differences in the proportion of males and females among the groups. Analysis of variance did reveal significant differences in age and education level for the TBI groups compared to the healthy groups. Multiple comparison testing revealed that the healthy subjects were significantly younger, more educated, and had higher IQ than the TBI groups. However, no difference in age, education level, and intelligence was found between TBI‐C and TBI‐M groups.

**Table 1:** Demographic characteristics of the participants

Group	*N*	Gender (% male)	Age (years)	Education (years)	Intelligence (IQ value)
H‐C	40	50	25.38 ± 3.32	15.78 ± 2.11	114.53 ± 5.64
H‐M	40	50	25.60 ± 4.40	15.58 ± 2.24	113.13 ± 5.41
TBI‐C	40	55	29.28 ± 8.11	9.06 ± 3.57	75.45 ± 7.35
TBI‐M	30	60	30.90 ± 7.03	8.27 ± 3.32	79.03 ± 10.07

H‐C, Healthy Control; H‐M, Healthy Malingering; TBI‐C, traumatic brain injury‐control; TBI‐M, traumatic brain injury‐malingering.

### 3.2 FGMT results

Between groups Kruskal–Wallis one‐way ANOVA for the easy item score, hard item score, and total score on the FGMT were all significant (each *p *<* *.01). Post hoc analysis revealed that the malingering groups performed significantly worse than the control groups. There was no difference in easy item score, hard item score, or total score between the H‐M and TBI‐M groups (*p = *.20, .08, and .05, respectively) or between the TBI‐C and the H‐C group (*p = *.44, .63, and .68, respectively). The results for each group on the FGMT are presented in Table 2.

**Table 2:** Forced‐choice Graphics Memory Test results in H‐C, H‐M, TBI‐C, TBI‐M

	Group	Mean ± *SD*	Mean rank	Kruskal–Wallis	*p*‐Value
Easy item score	H‐C	12.00 ± 0.00	113.50	109.35	<.01
	H‐M	8.15 ± 2.65	42.00^a^		
	TBI‐C	11.70 ± 0.56	101.83^b^		
	TBI‐M	7.87 ± 2.21	34.40^c^^,^ d		
Hard item score	H‐C	11.88 ± 0.34	118.69	119.99	<.01
	H‐M	4.00 ± 1.45	37.80^a^		
	TBI‐C	10.83 ± 1.65	102.00^b^		
	TBI‐M	3.70 ± 1.80	32.85^c^^,^ d		
Total score	H‐C	23.88 ± 0. 34	119.81	120.69	<.01
	H‐M	12.15 ± 3.03	37.34^a^		
	TBI‐C	22.53 ± 1.96	101.19^b^		
	TBI‐M	11.57 ± 3.05	33.05^c^^,^ d		

H‐C, Healthy Control; H‐M, Healthy Malingering; TBI‐C, traumatic brain injury‐control; TBI‐M, traumatic brain injury‐malingering.

a *p *<* *.01, H‐C versus H‐M.

b *p *<* *.01, H‐M versus TBI‐C.

c *p *<* *.01, H‐C versus TBI‐M.

d *p *<* *.01, TBI‐C versus TBI‐M.

### 3.3 Classification accuracy

**Figure 2:**
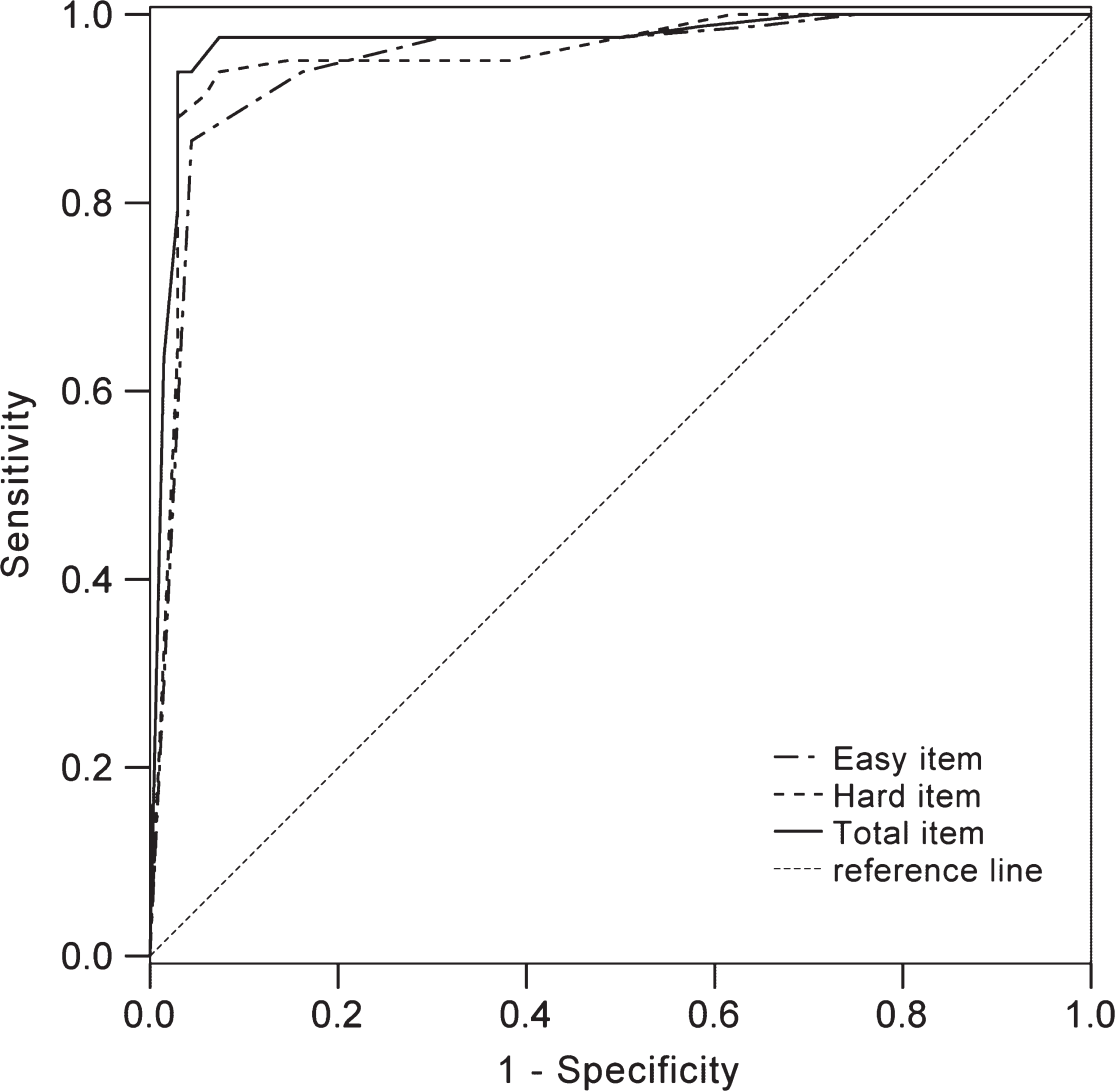
Receiver operating characteristic curve for the Forced‐choice Graphics Memory Test indices

Receiver operating characteristic analysis was performed to measure the classification ability of the three FGMT indices (easy item, hard item, and total score). See Figure 2. The area under the curve (AUC) for the total score was the highest (AUC = 0.97, 95% CI = 0.94–1.00), followed by the hard item score (AUC = 0.96, 95% CI = 0.92–0.99), and the easy item score (AUC = 0.95, 95% CI = 0.91–0.99), indicating that the total score has the highest classification ability in differentiating malingering from nonmalingering.

The sensitivity and specificity of various cutoff scores on easy items, hard items, and total score are presented in Table 3. Cutoff scores for each index were calculated by combining effects of sensitivity and specificity for each measure. A total score cutoff of less than 18 correctly categorized 100% of H‐C, 95% of H‐M, 95% of TBI‐C, and 93.3% of TBI‐M participants. A hard item score cutoff of less than 6 identified 95% of H‐C and H‐M, 90% of TBI‐C, and 93.3% of TBI‐M participants. An easy item score cutoff of less than 11 identified 97.5% of H‐C, 90% of H‐M, 95% of TBI‐C, and 83.3% of TBI‐M participants.

**Table 3:** The sensitivity and specificity rates in Forced‐choice Graphics Memory Test cutoff scores

Cutoff	Easy item score		Hard item score		Total score	
	Sens.	Spec.	Sens.	Spec.	Sens.	Spec.
≤2	1.00	0.00	1.00	0.21	1.00	0.00
3	1.00	0.09	1.00	0.38	1.00	0.00
4	1.00	0.12	0.95	0.62	1.00	0.00
5	1.00	0.16	0.95	0.85	1.00	0.02
**6**	1.00	0.25	**0.94**	**0.93**	1.00	0.04
7	0.99	0.35	0.92	0.94	1.00	0.06
8	0.98	0.50	0.89	97	1.00	0.09
9	0.98	0.69	0.87	0.97	1.00	0.13
10	0.94	0.84	0.82	0.97	1.00	0.24
**11**	**0.87**	**0.96**	0.65	0.97	1.00	0.29
12	0.00	1.00	0.00	1.00	0.99	0.41
13					0.98	0.50
14					0.98	0.62
15					0.98	0.75
16					0.98	0.93
17					0.94	0.96
**18**					**0.94**	**0.97**
19					0.93	0.97
20					0.89	0.97
21					0.85	0.97
22					0.79	0.97
23					0.63	0.99
24					0.00	1.00

Sensitivity is defined as the percentage of participants in the TBI‐M and H‐M groups that were correctly identified as responding invalidly. Specificity is defined as the percentage of participants in the TBI‐C and Healthy Control groups that were correctly identified as responding validly.

The bold values were highlighted to remind readers that these are the values when cutoff score is less than 18 of the total items, less than 6 of the easy hard items, and less than 11 of the easy items, respectively.

Predictive power was calculated using derivations on Bayes' theorem. For various research and clinical use, positive predictive value (PPV) and negative predictive value (NPV) were calculated for a range of base rates of invalid effort spanning 10% to 50%. Depending on the base rate used, PPV and NPV ranged from 0.18 to 0.98 and 0.5 to 1.0, respectively (Table 4).

**Table 4:** The predictive power for different base rates in Forced‐choice Graphics Memory Test cutoff scores

Cutoff	SN	SP	BR = 0.1		BR = 0.2		BR = 0.3		BR = 0.4		BR = 0.5	
			PPV	NPV	PPV	NPV	PPV	NPV	PPV	NPV	PPV	NPV
13	0.98	0.5	0.18	1.00	0.33	0.99	0.46	0.98	0.57	0.96	0.66	0.96
14	0.98	0.62	0.22	1.00	0.39	0.99	0.53	0.99	0.63	0.97	0.72	0.97
15	0.98	0.75	0.30	1.00	0.49	0.99	0.63	0.99	0.72	0.97	0.80	0.97
16	0.98	0.93	0.61	1.00	0.78	0.99	0.86	0.99	0.90	0.98	0.93	0.98
17	0.94	0.96	0.72	0.99	0.85	0.98	0.91	0.97	0.94	0.94	0.96	0.94
18	**0.94**	**0.97**	0.78	0.99	0.89	0.98	0.93	0.97	0.95	0.94	**0.97**	**0.94**
19	0.93	0.97	0.78	0.99	0.89	0.98	0.93	0.97	0.95	0.93	0.97	0.93
20	0.89	0.97	0.77	0.99	0.88	0.97	0.93	0.95	0.95	0.90	0.97	0.90
21	0.85	0.97	0.76	0.98	0.88	0.96	0.92	0.94	0.95	0.87	0.97	0.87
22	0.79	0.97	0.75	0.98	0.87	0.95	0.92	0.92	0.95	0.82	0.96	0.82
23	0.63	0.99	0.88	0.96	0.94	0.91	0.96	0.86	0.98	0.73	0.98	0.73

SN, sensitivity; BR, base rate; SP, specificity; RC, remaining cases = 1‐BR; PPV, positive predictive value, NPV, negative predictive value.

PPV = (SN × BR)/[(SN × BR) + (1 − SP) × RC].

NPV = (SP × RC)/[(SP × RC) + (1 − SN) × BR].

The bold values were highlighted to remind readers that these are the values when cutoff score is less than 18 of the total items, less than 6 of the easy hard items, and less than 11 of the easy items, respectively.

### 3.4 Test–retest reliability and internal consistency of the FGMT

We next examined the test–retest reliability and internal consistency of the FGMT. Using both TBI groups, we found strong 1 week test–retest reliability on all FGMT scores, including easy item score (*r *=* *.95, *p *<* *.01), hard item score (*r *=* *.98, *p *<* *.01), and total score (*r *=* *.99, *p *<* *.01). It should be noted that test–retest reliability of total score were higher in the TBI‐C (*r *=* *.97, *p *<* *.01) than in the TBI‐M group (*r *=* *.91, *p *<* *.01).

Internal consistency was assessed using Cronbach's alpha coefficients. The Cronbach's alpha coefficients were .93, .82, and .91 for the total 24 items, 12 easy items, and 12 hard items, respectively. The reliability of the test was also assessed by Guttman Split‐Half and Spearman‐Brown Split‐Half tests, and the coefficients were .79 and .84, respectively.

### 3.5 Convergent validity of the FGMT

Correlations were performed to investigate the relationships between the FGMT and the BFDMT. The easy item score (*r *=* *.68, *p *<* *.01), hard item score (*r *=* *.87, *p *<* *.01), and total score (*r *=* *.92, *p *<* *.01) of the FGMT were all positively correlated with the corresponding score of BFDMT.

### 3.6 Demographic characteristics and the FGMT

The participants of each TBI group were divided into groups based on education (<10 years; ≥10 years), gender, age (≤24 years, 25–34 years, ≥35 years), and intelligence (IQ < 70; IQ ≥ 70). In both the TBI‐C and TBI‐M groups, no significant differences in FGMT scores were found between education, gender, age, or intelligence groups (all *p *>* *.05, Table**** 5, 6, 7), suggesting FGMT scores are not related to these variables.

**Table 5:** Forced‐choice Graphics Memory Test scores in different subgroups of TBI‐C

Demographic variables	*N* (%)	Easy item score	Hard item score	Total score
Gender				
Male	22 (55.0)	11.64 ± 0.66	11.05 ± 1.43	22.68 ± 1.91
Female	18 (45.0)	11.78 ± 0.43	10.56 ± 1.89	22.33 ± 2.06
Age (years)				
≤24	15 (37.5)	11.93 ± 0.26	11.00 ± 1.36	22.93 ± 1.39
25–34	13 (32.5)	11.54 ± 0.66	10.69 ± 1.80	22.23 ± 2.20
≥35	12 (30.0)	11.58 ± 0.67	10.75 ± 1.91	22.33 ± 2.35
Education (years)				
<10	18 (45.0)	11.72 ± 0.58	10.89 ± 1.61	22.61 ± 1.82
≥10	22 (55.0)	11.68 ± 0.57	10.77 ± 1.72	22.45 ± 2.11

**Table 6:** Forced‐choice Graphics Memory Test scores in different subgroups of TBI‐M

Demographic variables	*N* (%)	Easy item score	Hard item score	Total score
Gender				
Male	18 (60.0)	8.28 ± 2.42	3.72 ± 1.90	12.00 ± 3.38
Female	12 (40.0)	7.25 ± 1.77	3.67 ± 1.72	10.92 ± 2.47
Age (years)				
≤24	7 (23.3)	9.29 ± 0.95	3.57 ± 2.07	12.86 ± 2.73
25–34	11 (36.7)	7.45 ± 1.81	4.09 ± 2.12	11.55 ± 2.70
≥35	12 (40.0)	7.42 ± 2.78	3.42 ± 1.38	10.83 ± 3.49
Education				
<10 years	20 (66.7)	7.75 ± 2.29	4.10 ± 1.90	11.85 ± 3.25
≥10 years	10 (33.3)	8.10 ± 2.13	2.90 ± 1.37	11.00 ± 2.11

**Table 7:** Forced‐choice Graphics Memory Test scores in different intelligence subgroups of traumatic brain injury

	IQ < 70(*N *=* *14)	IQ ≥ 70(*N *=* *56)
Easy item score	10.14 ± 1.79	10.04 ± 2.57
Hard item score	7.79 ± 3.66	7.77 ± 4.04
Total item score	17.93 ± 5.02	17.80 ± 6.25

## 4 Discussion

In this study, we developed a new PVT, the FGMT, and determined the ability of the FGMT to assess feigning. We found that FGMT performance accurately differentiated group performance with regard to effort status for both TBI and normal samples. When identifying invalid responses with the cutoff points of less than 11 of the easy items, less than six of the hard items, and less than 18 of the total items, respectively, total score cutoff produced the greatest classification accuracy of differentiating invalid responders from valid responders in both TBI and healthy groups. A total score cutoff of less than 18 was able to correctly categorize 100% of H‐C, 95% of H‐M, 95% of TBI‐C, and 93.3% of TBI‐M participants. Although this study was conducted with a base rate of poor effort of 50% in control participants and 42% in TBI participants, through an examination of PPV and NPV, we were able to demonstrate that the FGMT cutoff of less than 18 performs quite well across a variety of base rates. PPV ranged from 0.78 at a base rate of 10% invalid effort to 0.97 at a base rate of 50% poor effort. NPV remained extremely high across examined base rates, ranging from 0.94 to 0.99. Interestingly, the hard items had better classification accuracy than the easy items, which was inconsistent with what was observed in digital memory test (Liu et al., 2001). Forced‐choice digital memory test has been shown to be quite easy even for individuals with severe TBI and cognitive dysfunction, and therefore TBI patients should not make a large number of mistakes on the easy items (Guilmette, Whelihan, Sparadeo, & Buongiorno, 1994; Iverson & Binder, 2000). Accordingly, the easy digital items usually show higher classification accuracy than hard items (Liu et al., 2001). This discrepancy may due to the different sample capacity, as well as the different stimuli, as graphics are more on an intuitive basis than digits.

Various factors, such as education, intelligence, age and severity of injury, may affect the performance validity and subsequently reduce the value and reliability of PVT. The TOMM showed high reliability when considering the influence of age, education, psychiatric conditions, and cognitive impairment (Ashendorf, Constantinou, & McCaffrey, 2004; Gunner, Miele, Lynch, & McCaffrey, 2012; Iverson, Le Page, Koehler, Shojania, & Badii, 2007; Moser et al., 2007; Rees, Tombaugh, & Boulay, 2001; Teichner & Wagner, 2004). However, the administration time of two learning trials (Trials 1, 2) of TOMM is approximately 15 min (Lynch, 2004), and the retention trial takes 15–20 min. Similarly, the FGMT does not appear to be influenced by education, gender, age, or intelligence. Even people with a low level of education were able to complete the test without any difficulty. Additionally, The FGMT is easy to administer and time‐efficient (it only take 5–10 min to complete).

A potential limitation of this study is the inclusion of only subjects classified as “definite malingering” or “not malingering,” but not the “probable malingering” or “possible malingering,” and only one clinical sample of TBI patients was detected, leading to high sensitivity and specificity. However, the empirical cutoff scores of PVT are developed based on the “purity” of the control and malingering groups (Greve & Bianchini, 2004). Further research is needed to detect the cross‐validation in different types of clinical presentations (stroke, dementia etc.).

Another potential limitation of the study is that we only included the patients with severe brain injury. Future research should address the influence of neurological (e.g., location, severity, or type of lesion) and psychological conditions on FGMT (Bigler, 2012; Larrabee, 2012). Additionally, the PVTs were administered at the beginning of the battery and order of PVTs was not counterbalanced. It is possible that this may have biased the results. Future studies should take care to investigate how the FGMT performs when it is administered in the middle or at the end of a testing battery. Convergent validity using a PVT of very different design (e.g., a nonforced‐choice test) should also be investigated in future.

In conclusion, we developed a reliable, simple measure, the FGMT, based on binomial theorem to identify malingering in patients with TBI and controls. This measure does not appear to be influenced by education, gender, age, or intelligence level. The FGMT has high classification accuracy, test–retest reliability, and internal consistency in a Chinese sample. Future studies are needed to replicate the present results in larger samples and other neurological and ethnic samples.

## Conflicts of Interest

The authors report no conflicts of interest.
